# Supplementary material for: A ML-Based Resource Allocation Scheme for Energy Optimization in 5G NR
Source: Sensors (Basel). 2025 Aug 12;25(16):4978. doi: 10.3390/s25164978 (PMC12389871; doi:10.3390/s25164978)
Supplement: Supplementary file 1 [file sensors-25-04978-s001.zip › sensors-3746251-supplementary.pdf]

sensors

Supplementary Material

The performance in every sleep state for each cell.

Table S1. Energy saving performance for n28 cell.

State	BW (MHz)	DL slots	UL slots	Relat. Power	PDSCH (kREs/s)	PDCCH (kREs/s)	DRB (bearer/s)	DRB in grid
0	3.60	0	10	0.02	0	0	0	0
1	9.38	1	9	0.13	0	752.4	156	3
2	9.38	2	8	0.22	0	1504.8	313	6
3	9.38	4	6	0.41	0	3189.6	664	13
4	9.38	6	4	0.60	0	4874.4	1015	20
5	9.38	8	2	0.79	0	6559.2	1366	27
6	9.38	10	0	0.99	0	8244.0	1717	34

[1] PDCCH throughput in n28 cell is always 0, because in our network model definition, this cell is just used for data traffic so we disabled the PDCCH signal.

Table S2. Energy saving performance for n78-1/3 cell.

State	BW (MHz)	DL slots	UL slots	Relat. Power	PDSCH (kREs/s)	PDCCH (kREs/s)	DRB (bearer/s)	DRB in grid
0	7.2	0	0	0.04	1152.0	0	0	0
1	39.3	1	1	0.14	1152.0	3416.4	711	14
2	39.3	2	1	0.24	1152.0	6832.8	1423	28
3	39.3	3	2	0.33	1152.0	10249.2	2135	43
4	39.3	4	2	0.43	1152.0	13665.6	2837	57
5	39.3	5	3	0.53	1152.0	17082.0	3558	71
6	39.3	6	4	0.62	1152.0	20498.4	4270	85

[2] The n78-1 and n78-3 cell have the same bandwidth assigned also the same sleep scheme, hence the performances are the same.

Table S3. Energy saving performance for n78-2 cell.

State	BW (MHz)	DL slots	UL slots	Relat. Power	PDSCH (kREs/s)	PDCCH (kREs/s)	DRB (bearer/s)	DRB in grid
0	7.2	0	0	0.08	1152.0	0	0	0
1	19.8	1	1	0.17	1152.0	1566.0	326	7
2	19.8	2	1	0.26	1152.0	3138.3	653	13
3	19.8	3	2	0.35	1152.0	4704.3	980	20
4	19.8	4	2	0.44	1152.0	6276.6	1307	26
5	19.8	5	3	0.53	1152.0	7943.4	1654	33
6	19.8	6	4	0.62	1152.0	9610.2	2002	40

 https://doi.org/10.3390/s25164978

To explain why choosing k factor as 10 for the KNN model in our paper, we present the Figure S1.

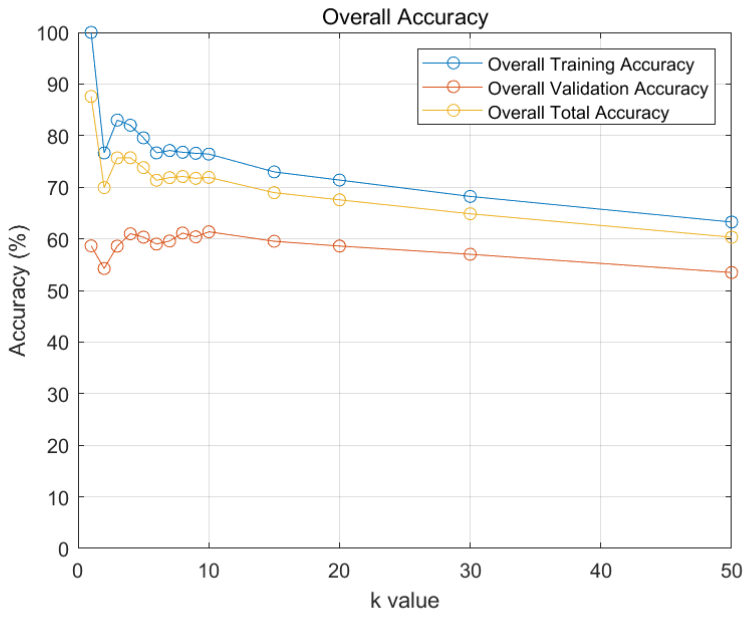

Figure S1. Overall Accuracy for KNN model with different k values.

The figure illustrates how the choice of k in the KNN algorithm impacts the model's performance across five different cells. Lower k values tend to overfit, while higher k values lead to underfitting. In our project, the optimal k value appears to be 4.

Detailed UE DRB usage distribution for every cell in worst scenario categorized by different user type.

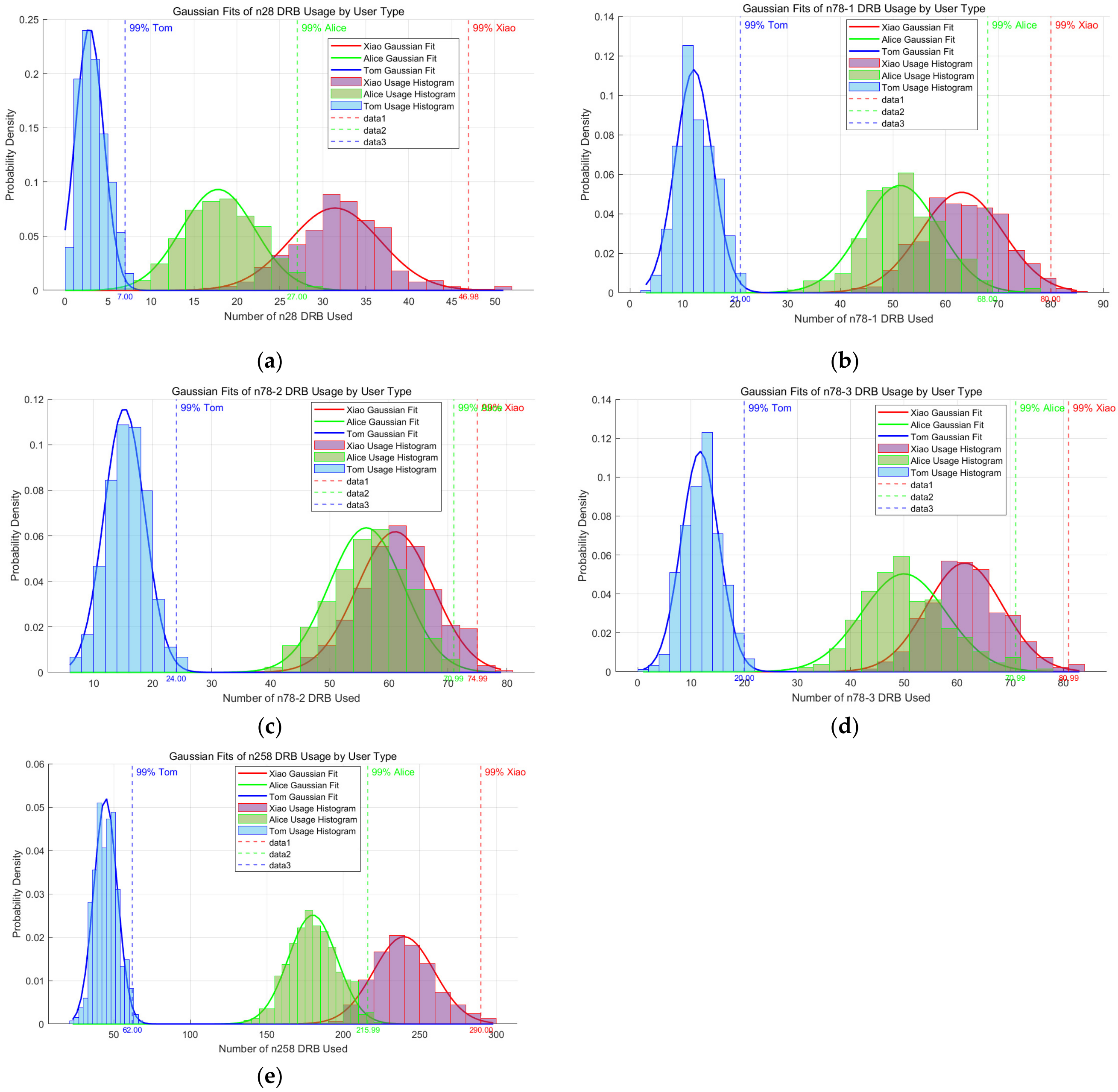

Figure S2. Distribution of DRB usage by different user types in worst scenario: (**a**) n28 cell; (**b**) n78-1 cell; (**c**) n78-2 cell; (**d**) n78-3 cell; (**e**) n258 cell.

Detailed Learning and validation curves of each cell.

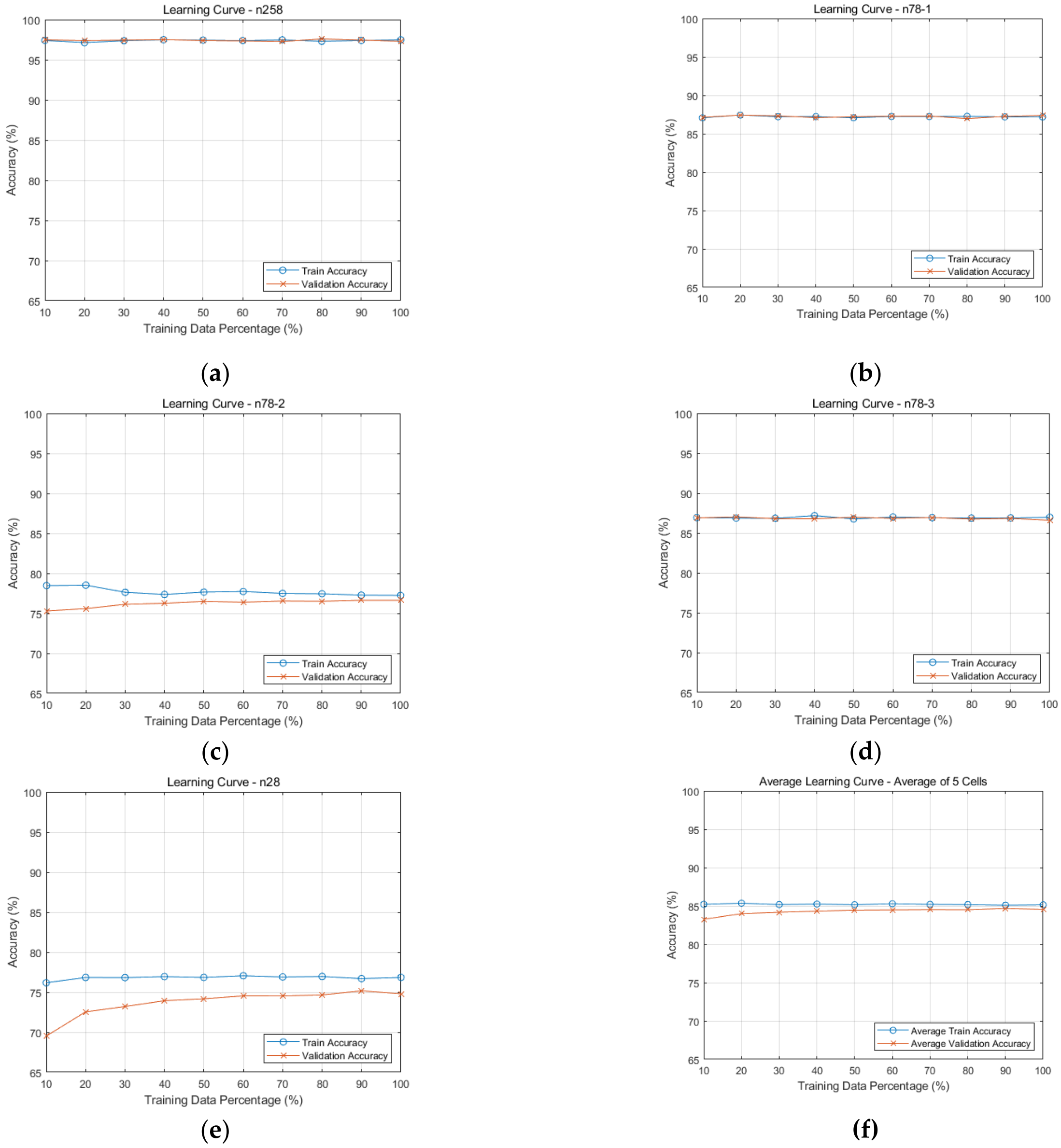

Figure S3. Learning and validation curve: (**a**) n258 cell; (**b**) n78-1 cell; (**c**) n78-2 cell; (**d**) n78-3 cell; (**e**) n28 cell; (**f**) average of 5 cells.

Detailed analysis of how MinLeafSize affects the performance of each cell.

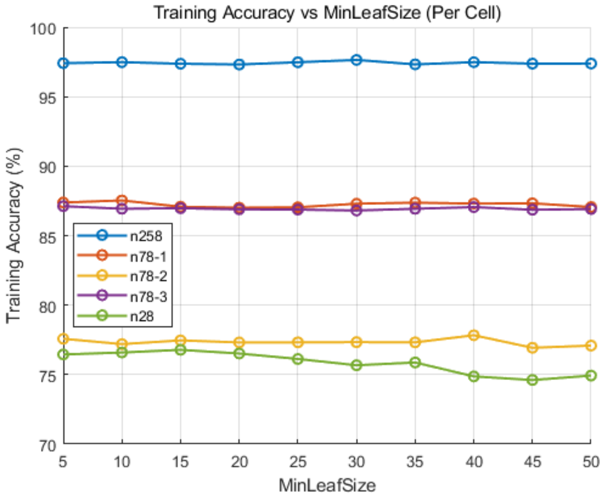

(**a**)

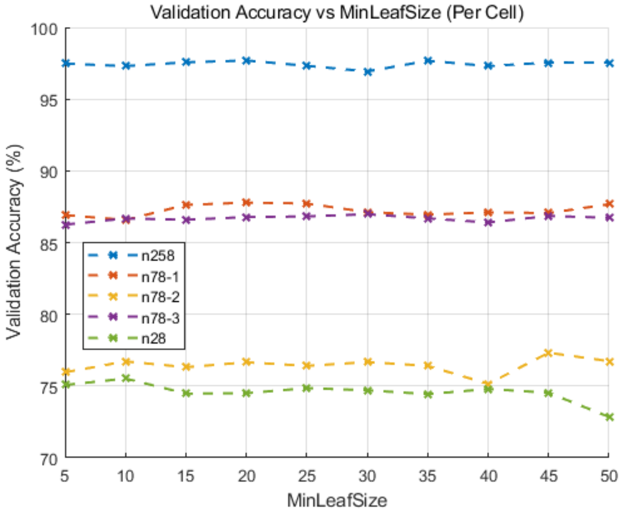

(**b**)

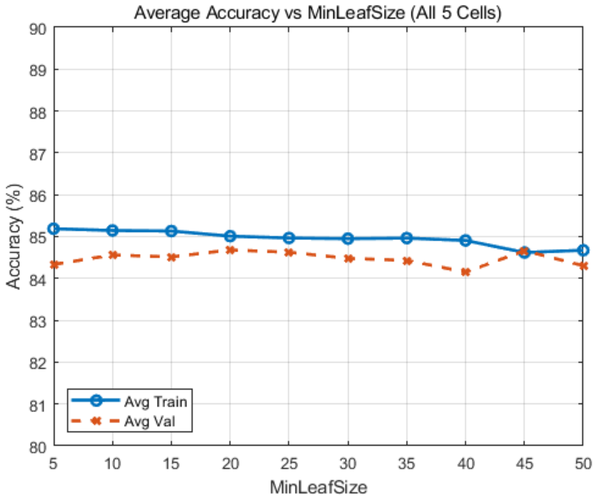

(**c**)

Figure S4. How MinLeafSize affects the performance of each cell: (**a**) training accuracy for each cell; (**b**) validation accuracy for each cell; (**c**) average training and validation accuracy of all cells.

Detailed analysis of how MaxNumSplits affects the performance of each cell.

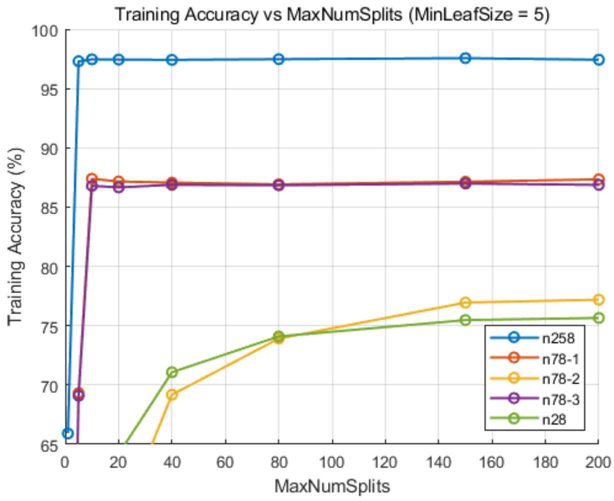

(**a**)

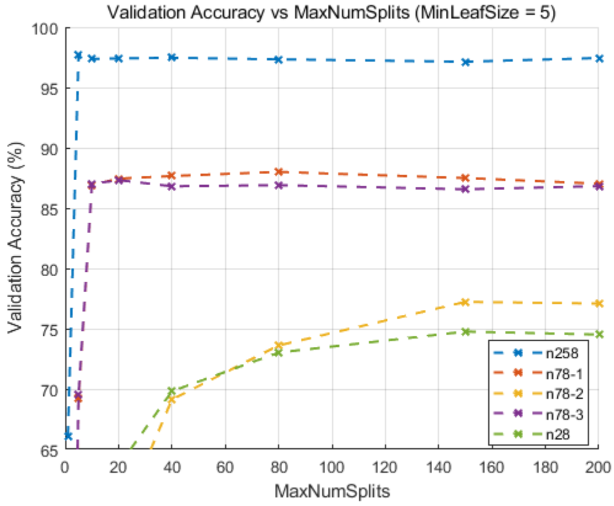

(**b**)

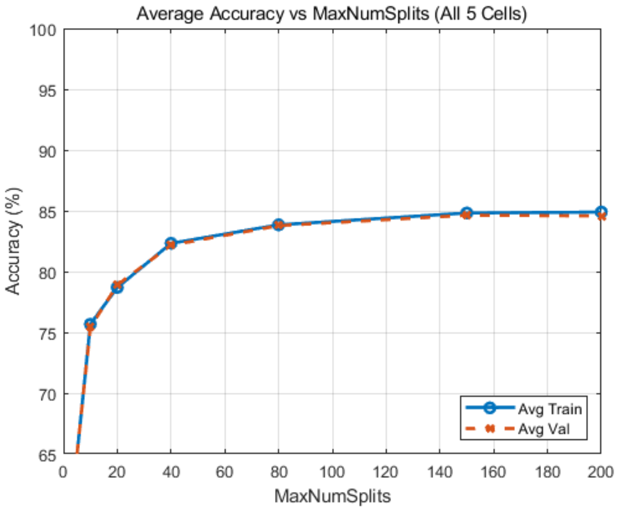

(**c**)

Figure S5. How MaxNumSplits affects the performance of each cell: (**a**) training accuracy for each cell; (**b**) validation accuracy for each cell; (**c**) average training and validation accuracy of all cells.

D etailed interpreted visual classification tree of each cell.

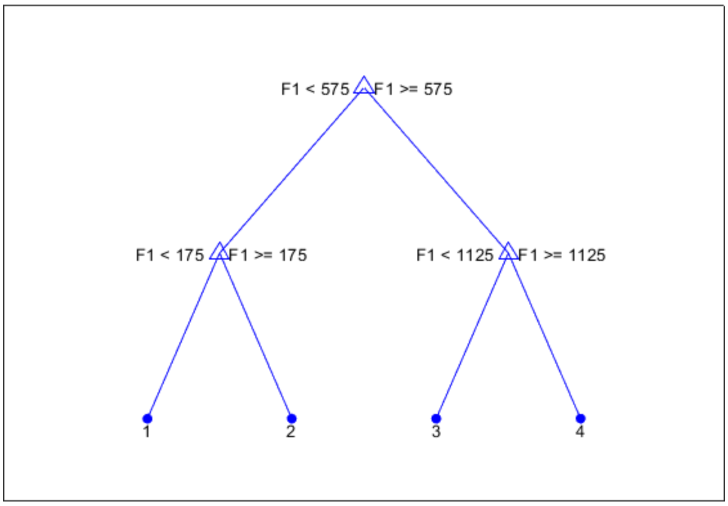

(**a**)

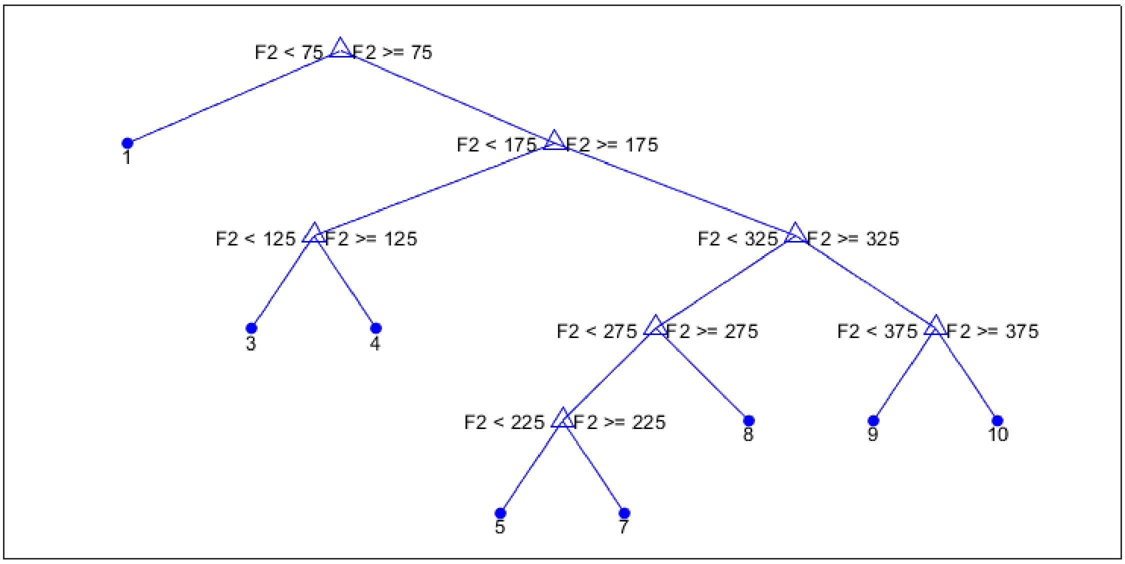

(**b**)

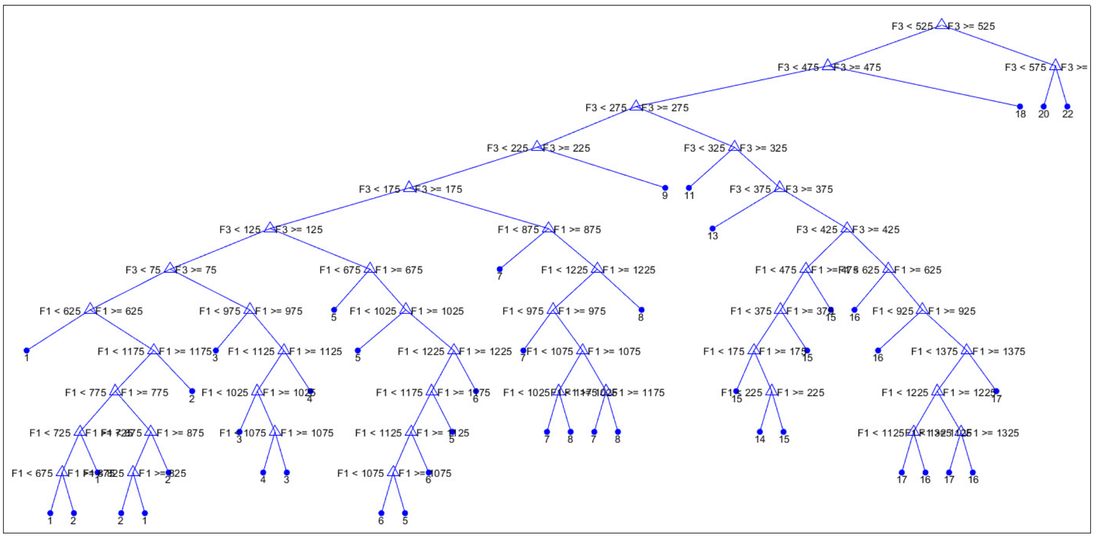

(**c**)

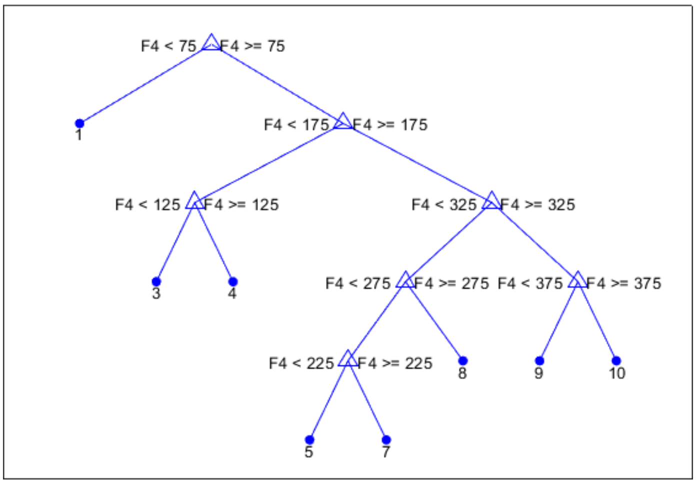

(**d**)

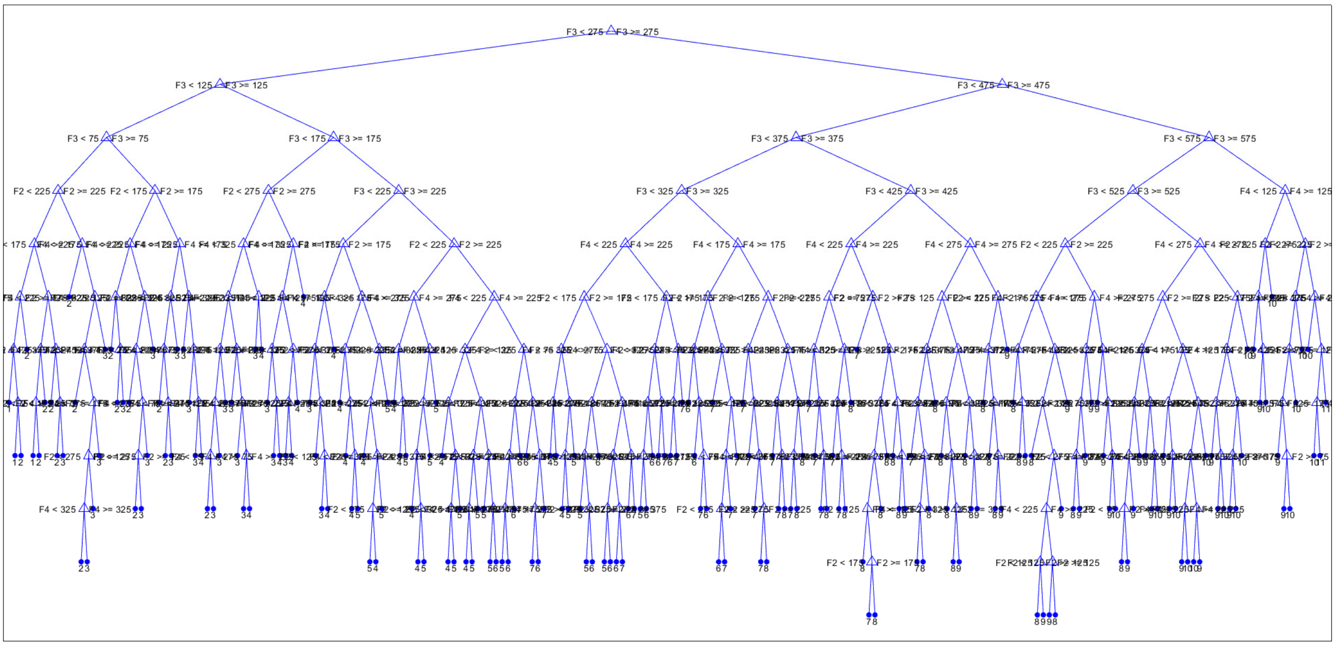

(**e**)

Figure S6. Interpreted visual classification tree of each cell: (**a**) n258 cell; (**b**) n78-1 cell; (**c**) n78-2 cell; (**d**) n78-3 cell; (**e**) n28 cell.

Detailed confusion matrix of each cell.

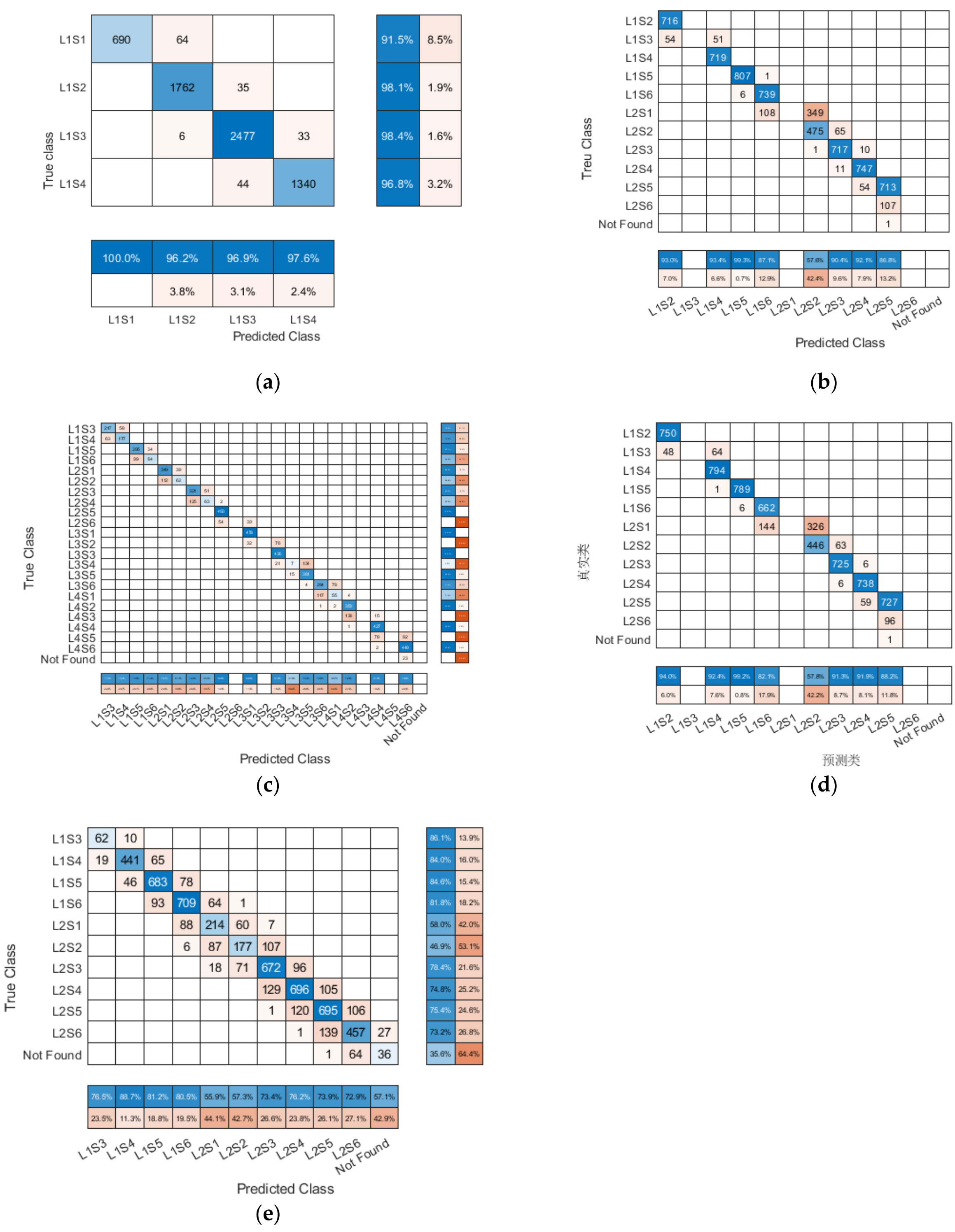

Figure S7. Confusion matrix: (**a**) n258 cell; (**b**) n78-1 cell; (**c**) n78-2 cell; (**d**) n78-3 cell; (**e**) n28 cell.
